# Low reliability of DNA methylation across Illumina Infinium platforms in cord blood: implications for replication studies and meta-analyses of prenatal exposures

**DOI:** 10.1186/s13148-022-01299-3

**Published:** 2022-06-28

**Authors:** Emilie Willoch Olstad, Hedvig Marie Egeland Nordeng, Geir Kjetil Sandve, Robert Lyle, Kristina Gervin

**Affiliations:** 1 Pharmacoepidemiology and Drug Safety Research Group, Department of Pharmacy, Faculty of Mathematics and Natural Sciences, University of Oslo, Oslo, Norway; 2 PharmaTox Strategic Research Initiative, Faculty of Mathematics and Natural Sciences, University of Oslo, Oslo, Norway; 3 Department of Child Health and Development, Norwegian Institute of Public Health, Oslo, Norway; 4 Department of Informatics, Faculty of Mathematics and Natural Sciences, University of Oslo, Oslo, Norway; 5 Department of Medical Genetics, Oslo University Hospital and University of Oslo, Oslo, Norway; 6 Centre for Fertility and Health, Norwegian Institute of Public Health, Oslo, Norway; 7 Department of Research and Innovation, Division of Clinical Neuroscience, Oslo University Hospital, Oslo, Norway

**Keywords:** Epigenetic epidemiology, Epigenetics, EWAS, MoBa, MBRN, Validity, Replication, Reliability, Illumina Infinium platforms, Microarrays

## Abstract

**Background:**

There is an increasing interest in the role of epigenetics in epidemiology, but the emerging research field faces several critical biological and technical challenges. In particular, recent studies have shown poor correlation of measured DNA methylation (DNAm) levels within and across Illumina Infinium platforms in various tissues. In this study, we have investigated concordance between 450 k and EPIC Infinium platforms in cord blood. We could not replicate our previous findings on the association of prenatal paracetamol exposure with cord blood DNAm, which prompted an investigation of cross-platform DNAm differences.

**Results:**

This study is based on two DNAm data sets from cord blood samples selected from the Norwegian Mother, Father and Child Cohort Study (MoBa). DNAm of one data set was measured using the 450 k platform and the other data set was measured using the EPIC platform. Initial analyses of the EPIC data could not replicate any of our previous significant findings in the 450 k data on associations between prenatal paracetamol exposure and cord blood DNAm. A subset of the samples (*n* = 17) was included in both data sets, which enabled analyses of technical sources potentially contributing to the negative replication. Analyses of these 17 samples with repeated measurements revealed high per-sample correlations ($\stackrel{\mathrm{-}}{\text{R}}\hspace{0.17em}\approx$ 0.99), but low per-CpG correlations ($\stackrel{\mathrm{-}}{\text{R}}$ ≈ 0.24) between the platforms. 1.7% of the CpGs exhibited a mean DNAm difference across platforms > 0.1. Furthermore, only 26.7% of the CpGs exhibited a moderate or better cross-platform reliability (intra-class correlation coefficient ≥ 0.5).

**Conclusion:**

The observations of low cross-platform probe correlation and reliability corroborate previous reports in other tissues. Our study cannot determine the origin of the differences between platforms. Nevertheless, it emulates the setting in studies using data from multiple Infinium platforms, often analysed several years apart. Therefore, the findings may have important implications for future epigenome-wide association studies (EWASs), in replication, meta-analyses and longitudinal studies. Cognisance and transparency of the challenges related to cross-platform studies may enhance the interpretation, replicability and validity of EWAS results both in cord blood and other tissues, ultimately improving the clinical relevance of epigenetic epidemiology.

**Supplementary Information:**

The online version contains supplementary material available at 10.1186/s13148-022-01299-3.

## Background

Epigenetics entails modifications of the DNA that can impact gene expression, but does not involve changes in the underlying DNA sequence. The most commonly studied epigenetic modification is DNA methylation (DNAm), which occurs at cytosine bases of cytosine–phosphate–guanine dinucleotide sites (CpGs). DNAm can be impacted by the DNA sequence, as well as environmental influences [1–4]. There is an increasing interest in the role of epigenetics within epidemiology. Several pharmacoepidemiological studies have reported an association between prenatal psychotropic or analgesic medication exposure and neurodevelopmental outcomes in the offspring [5–13]. Furthermore, multiple epigenome-wide association studies (EWASs) have identified DNAm changes associated with medication exposure during pregnancy (e.g. valproic acid, antidepressants and paracetamol) [14–20]. Recently, we found an association between cord blood DNAm and prenatal long-term exposure to paracetamol in children with attention-deficit/hyperactivity disorder (ADHD) [21]. These initial findings may suggest that DNAm is involved in the relationship between prenatal medication exposure and adverse neurodevelopmental outcomes [3, 4].

Despite a growing interest in epigenetics, and an increasing number of published EWASs, there are several critical biological and technical challenges in epigenetic epidemiology, which have important implications for the interpretation, validity and clinical translation of the findings [1, 22, 23]. One key challenge is the paucity in the replication of findings. For instance, two systematic literature reviews on the association of offspring epigenetic patterns with medication use [20] and maternal well-being in pregnancy [24] uncovered largely inconsistent findings. These reviews suggest multiple origins of the discrepant results, such as small sample sizes resulting in low statistical power and poor study designs [20, 24]. The majority of EWASs are based on DNAm data generated using the Illumina Infinium HumanMethylation BeadChip platforms, including the 27 k (*n* > 27,000 CpGs), 450 k (*n* > 450,000 CpGs) and the EPIC arrays (*n* > 850,000 CpGs) [25]. Recent studies have elucidated technical aspects related to the Infinium platforms, which have significant influences on the analyses and interpretation of results. These studies have shown significant per-CpG differences and poor per-CpG correlation both within [26–35] and across [31, 32, 36–40] microarray platforms, which challenges combined analyses of DNAm data from both platforms (e.g. [41–45]). In cord blood, the median correlation of individual CpGs across platforms was only 0.24 [37]. Furthermore, 2.4% of the CpGs exhibited a mean difference in measured DNAm level between the platforms ≥ 0.1 [37], on the same order as the low effect sizes often observed within epigenetic epidemiology [1, 22, 46]. Furthermore, only 18.0% of CpGs in adult whole-blood exhibit a moderate or better reliability across platforms (intra-class correlation coefficient [ICC] ≥ 0.5) [31]. The technical aspects contributing to low reliabilities of CpGs may affect the power of EWASs [28, 47]. Consequently, poor concordance of measured DNAm levels across platforms may impact both the replicability and validity of EWAS results.

In an ongoing study, we aim to replicate and expand our previous findings showing associations between long-term prenatal exposure to paracetamol (≥ 20 days) and DNAm in children with ADHD [21]. Analyses of DNAm data generated from a larger number of samples selected from the same cohort using the Infinium EPIC platform find no significant CpGs associated with paracetamol exposure. Accordingly, we fail to replicate any of our previous significant findings [21]. Examining a subset of samples with repeated measurements in both data sets has enabled a thorough investigation of potential technical origins of the negative replication. Our findings could not explain the failure to replicate our previous results, but are still important for replication EWASs, as well as studies combining DNAm from different Infinium platforms, such as longitudinal studies or meta-analyses.

## Results

### Lack of replicability may originate from several technical sources

This study is based on a subset of samples (*n* = 17) included in two datasets and consists of repeated measurements using the Infinium 450 k and EPIC platforms. The samples were selected from the Norwegian Mother, Father and Child Cohort Study (MoBa). In the data set assessed on the 450 k platform (*n* = 384 samples), we have previously published associations between prenatal exposure to paracetamol and DNAm differences in children with ADHD [21]. Analysis of the second data set (*n* = 261 samples), which was designed to expand on these findings using the EPIC platform, has failed to replicate our previous findings (data not shown). This prompted a thorough investigation of whether technical aspects of the Infinium platforms could explain the negative replication. Using a subset of samples with repeated measurements from both studies (*n* = 17 samples), we conducted systematic analyses to assess the integrity and reliability of the DNAm data between the Infinium platforms.

#### The DNAm data separate into clusters explained by microarray platforms

**Fig. 1:**
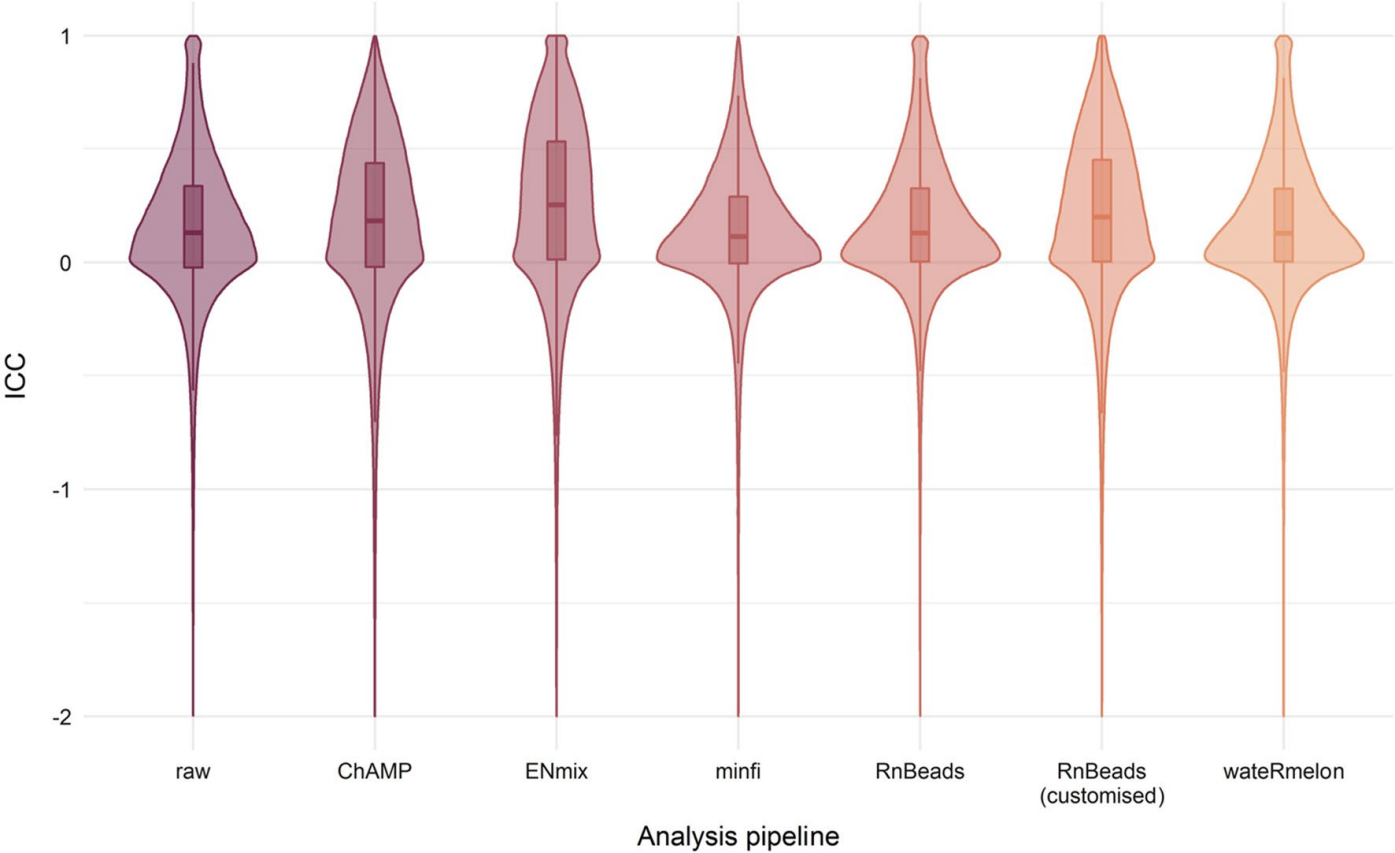
Overview of the ICC distribution computed from raw data and from data pre-processed using the default settings of five common EWAS analysis pipelines. Additionally, we included one common analysis pipeline (“RnBeads (customised)”, including the normalisation methods *ENmix.oob* and *BMIQ*). All pipelines examined also exhibited ICCs lower than –2, but these were removed from the illustration for visualisation purposes. The default settings of each analysis pipeline are detailed in Additional file 1: Table S1

We performed stringent quality control, normalisation and probe filtering procedures of the DNAm data from the two data sets containing the samples with repeated measurements, to minimise technical variation related to pre-processing of the data. First, we examined DNAm data measured for a set of genotyping probes on each platform (*n* = 59 probes). Clustered heatmaps of DNAm values at these genotyping probes showed that the repeated cross-platform measurements of each sample grouped together and hence, excluded potential mix-up of samples (Additional file 1: Fig. S1). Second, we examined whether pre-processing steps such as background and probe-type correction impacted the cross-platform concordance. To do this, we used the intra-class correlation coefficient (ICC), which equals 1 if there is perfect per-CpG concordance between the measured DNAm in the 450 k and EPIC data sets. Generally, an ICC < 0.5 is considered poor [48, 49]. We computed the ICCs after pre-processing the 450 k and EPIC data sets separately, using the default settings of five commonly used pre-processing pipelines *ChAMP* [50, 51], *ENmix* [34], *minfi* [52], *RnBeads* [53] and *wateRmelon* [54] (Additional File 1: Table S1). We also included one pipeline commonly reported in the literature, namely *RnBeads* with the background and probe-type corrections *ENmix.oob* [34] and BMIQ [55], respectively. This analysis revealed that the *ENmix* pipeline exhibited larger ICCs than the other pipelines (Fig. 1). Therefore, we performed the rest of the analyses on data sets normalised using the default settings of the *ENmix* pipeline.

**Fig. 2:**
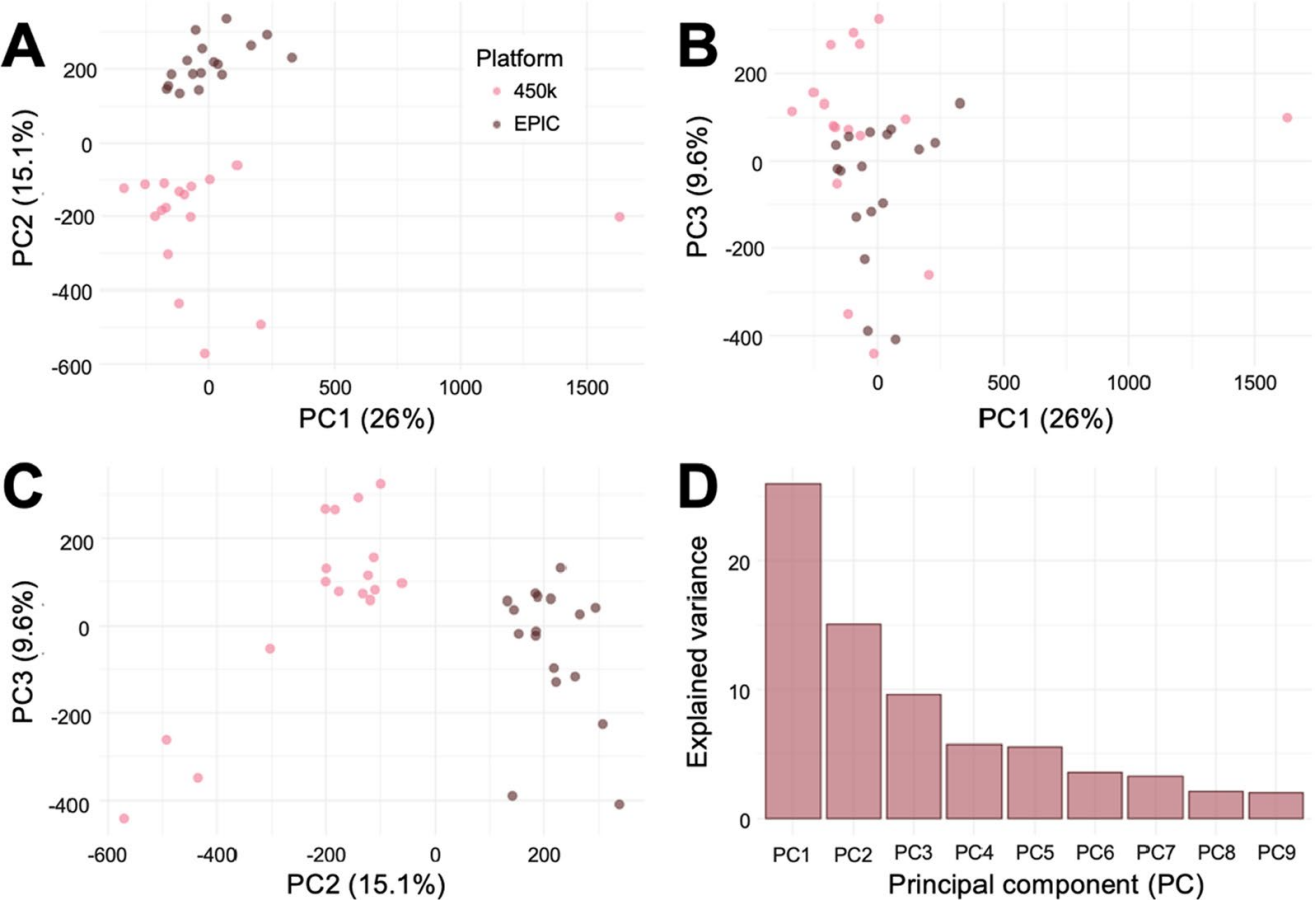
**(A–C)** Scatter plots of the first three principal components (PC1–3) from PCA of DNAm data from samples with repeated measurements (*n* = 17 samples) using the 450 k and EPIC platforms, and **(D)** a scree plot showing the amount of variance explained by the first nine PCs

Next, we performed principal component analysis (PCA) to explore technical variation in the DNAm data related to the 450 k and EPIC platforms. As expected, PCA revealed distinct clustering of samples corresponding to the 450 k and EPIC platforms (Fig. 2). Similar plots were observed when pooling all the available 450 k and EPIC samples (*n* = 628 samples; data not shown).

#### DNAm levels differ between the 450 k and EPIC platforms

**Fig. 3:**
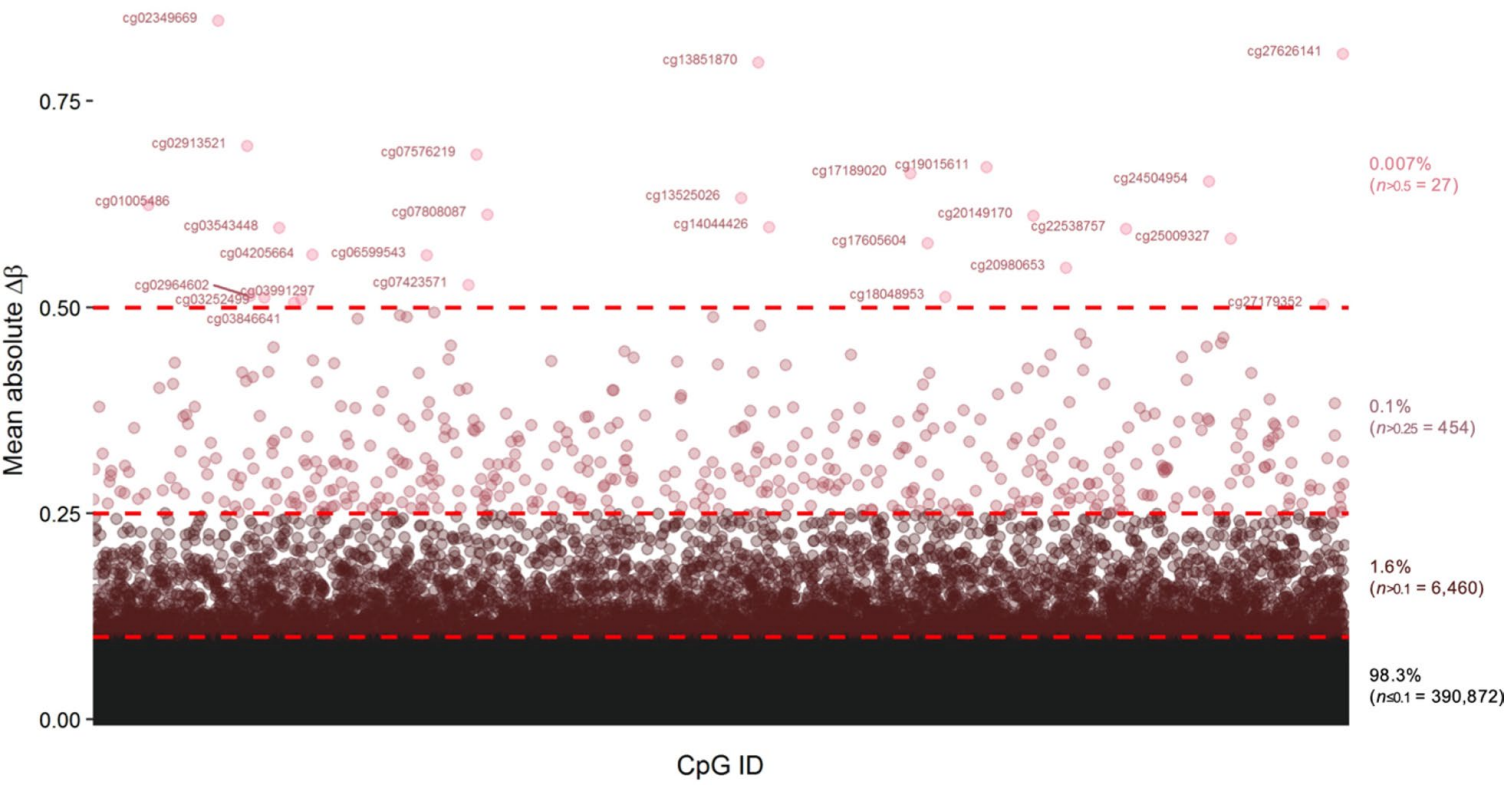
Mean absolute difference in measured DNA methylation (*Δβ*) per CpG, on the 450 k and EPIC platforms. Red dotted lines indicate a mean *Δβ* > 0.1, > 0.25 and > 0.5. Illumina CpG IDs are named if the mean *Δβ *> 0.5

To further investigate the dissimilarities between the 450 k and EPIC platforms, we computed the difference in and correlation of DNAm at overlapping CpGs on the two platforms (*n* = 397,813 CpGs). These analyses revealed small per-sample absolute differences in DNAm at overlapping CpGs between the two arrays (median ≈ 0.008 and mean ≈ 0.017 absolute DNAm differences). For 0.1% (*n* = 454) of CpGs, the mean DNAm difference over all replicates was > 0.25, while 0.007% (*n* = 27) of CpGs exhibited a mean DNAm difference > 0.5 (Fig. 3). These numbers are largely in line with previous studies, comparing differences in measured DNAm between the 450 k and EPIC arrays in cord blood [37], whole-blood [31, 32, 36, 37], placenta [38] and cartilage [39]. Furthermore, of the 27 CpGs with an absolute mean DNAm difference > 0.5, 5 of these CpGs also exhibited absolute mean DNAm difference > 0.5 in both cord blood [37], whole-blood [37], placenta [38] and cartilage [39] (Additional file 1: Fig. S2).

**Fig. 4:**
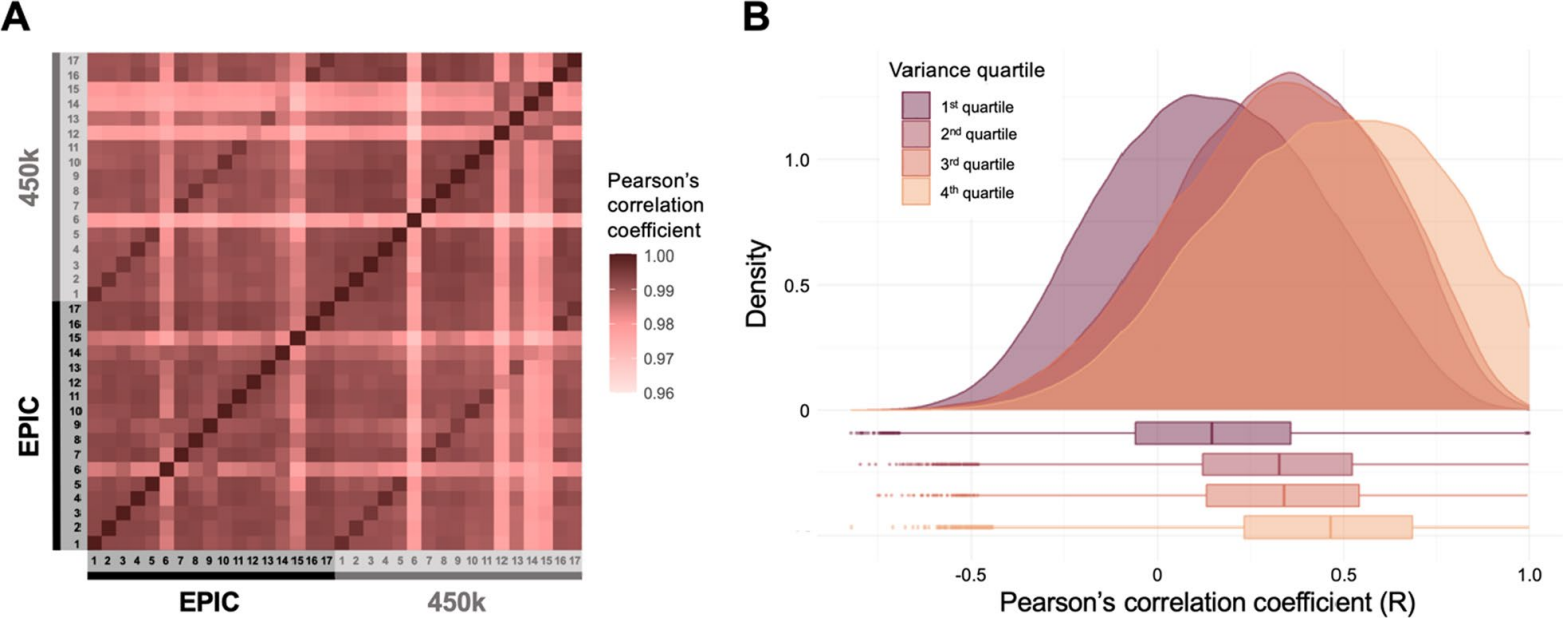
Pearson’s correlation coefficients of DNAm in replicates of the 450 k and EPIC platforms, for **(A)** per-sample correlations in a correlogram, and **(B)** per-CpG correlations as distributions stratified by variance quartiles, based on the variance of the respective CpGs on the EPIC platform

We observed a high per-sample correlation of DNAm between the platforms, both when comparing replicates, and when comparing two independent samples across the platforms (Fig. 4A). The median per-sample Pearson’s correlation coefficient was 0.996 and the mean was 0.992, with the lowest correlation between any two samples being 0.969 and the highest being 0.998. In contrast, the per-CpG correlations of measured DNAm between the platforms were significantly lower: the median correlation was 0.237 and the mean was 0.238, with the lowest correlation being -0.822 and the highest being 1.00 (Fig. 4B). The per-CpG correlation appeared to be related to the variance of each CpG, which were similar for both platforms; CpGs with high correlation also exhibited larger variance (Fig. 4B). The high per-sample correlation, low per-CpG correlation, and the relationship between CpG variance and correlation, have previously been reported for cord blood [37], and multiple other tissues [31, 32, 36–39].

#### Few CpGs are reliable between the 450 k and EPIC platforms

**Fig. 5:**
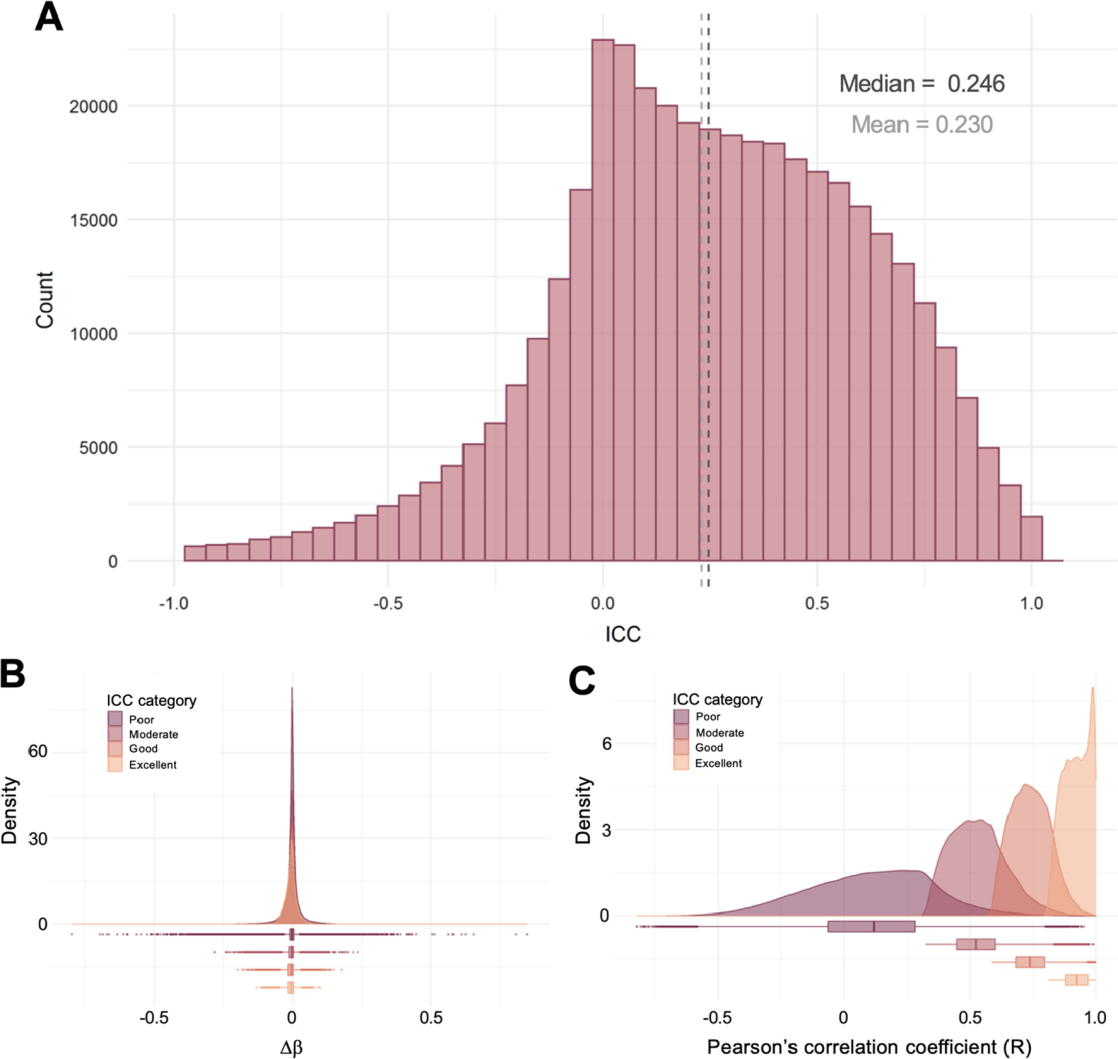
**(A)** Histogram of the ICCs computed from the 17 samples assessed on both the 450 k and EPIC platforms. **(B)** Density distribution of mean difference in DNAm level, stratified by ICC category. **(C)** Density distribution of Pearson’s correlation coefficient, stratified by ICC category. The ICC categories are defined as follows: poor: ICC < 0.5; moderate: 0.5 ≤ ICC < 0.75; good: 0.75 ≤ ICC < 0.9; and excellent: ICC ≥ 0.9. The dark grey, dotted line indicates the median ICC, and the light grey, dotted line indicates the mean ICC. Outlying CpGs with ICCs less than the mean ICC minus three standard deviations were removed for visualisation purposes, but were included for summary statistic calculations

In order to examine concordance of cross-platform DNAm levels, we assessed the reliability of the CpGs, reflecting both correlation and agreement. To do this, we computed the ICC, as previously suggested by Sugden et al*.* (2020) comparing cross-platform DNAm levels in adult whole-blood [31]. Overall, the ICCs of the overlapping CpGs were poor (median = 0.246 and mean = 0.230; Fig. 5A). Approximately 26.7% (*n* = 106,078) of the CpGs exhibited an ICC ≥ 0.5. This is similar to the findings of the recent study by Sugden et al*.* in adult whole-blood, where 18.0% of CpGs exhibited an ICC ≥ 0.5 [31]. Approximately 38.6% (*n* = 40,916) of the CpGs with an ICC ≥ 0.5 in the current study overlapped with the CpGs with an ICC ≥ 0.5 reported by Sugden et al*.* [31] (Additional File 2). The microarray type II probes exhibited slightly better ICCs and correlation coefficients than type I probes (Additional File 1: Fig. S3). Probes with poor ICCs and correlation coefficients appear more frequently in CpG islands (Additional File 1: Figs. S4 and S5), possibly due to an increased proportion of largely unmethylated CpGs in these regions (Additional File 1: Fig. S6).

Considering the poor CpG reliabilities, we investigated if the ICCs of the repeated measurements were higher than expected for two randomly paired samples. Therefore, we paired each EPIC sample with a randomly selected 450 k sample. The distribution of ICCs computed from the 17 repeated measurements (Fig. 5A) is significantly different from the ICC distributions computed from the 17 random 450 k-EPIC pairs (Kolmogorov–Smirnov test: *p* < 2.2*10^–16^; Additional file 1: Fig. S7). Furthermore, only a small percentage of the CpGs of the random pairs (2.4%–4.8%) exhibited an ICC ≥ 0.5, which are significantly different proportions from the ICCs of the repeated measurements (Pearson’s Chi-squared test: *p* < 2.2*10^–16^).

#### The ICC reflects both correlation and agreement across microarray platforms

To investigate if the ICCs reflect both agreement and correlation across platforms, we examined the distribution of mean differences in DNAm and Pearson’s correlation coefficients, for each of four ICC categories: poor (ICC < 0.5), moderate (0.5 ≤ ICC < 0.75), good (0.75 ≤ ICC < 0.9) and excellent (ICC≥ 0.9) [48]. The distribution of mean differences in DNAm is relatively similar between the ICC categories. However, there are far more of the poor CpGs displaying large differences in mean DNAm levels across platforms compared to the other ICC categories (Fig. 5B). In contrast, the correlation coefficient increases with improving ICC category; the poor ICC category exhibits a wide range of low correlation coefficients (median ≈ 0.12), while the distribution of the correlation in the excellent category is highly skewed to the right (median ≈ 0.92). The moderate and good categories exhibit a wider range of correlation coefficients than the excellent CpGs, with a median of 0.52 and 0.74, respectively (Fig. 5C).

These observations demonstrate that the reliability of each CpG depends on both the correlation and the agreement between the two platforms [48]. An excellent CpG will have both a low mean difference in DNAm between platforms and a high correlation, explaining the small range in values of both the mean DNAm differences and the correlation coefficients. In contrast, a poor probe (including a larger range of ICCs) may exhibit an acceptable correlation but have a large mean DNAm difference (Additional file 1: Fig. S8). For instance, 685 of the 5407 CpGs with an R≥ 0.9 nevertheless have an ICC ≤ 0.9, with 22 CpGs even having a poor ICC (< 0.5). Furthermore, of the 395,286 CpGs with a mean DNAm difference ≤ 0.1, 289,327 exhibit a poor ICC (< 0.5). This is likely due to low correlations, as the median R for these CpGs is 0.12, while the median R was 0.59 for the 105,959 CpGs with a mean DNAm difference ≤ 0.1 and an ICC ≥ 0.5. Hence, the ICC better reflects reliability across platforms than either accuracy or correlation on their own.

#### The significant CpGs in the 450 k data have low reliabilities

We then asked if our failure to replicate the findings in our original study [21] could be explained by poor-performing probes, by examining the ICCs of the significant CpGs from the 450 k data set. The significant CpGs for the three group comparisons performed in the original study have median ICCs of 0.119, 0.122 and 0.135 (Additional file 1: Fig. S9). These reliabilities are low compared to the overall mean and median of the ICCs including all common CpGs across platforms.

## Discussion

Replication of association studies is important to ensure robust and valid findings. In an ongoing study, we aimed to replicate and expand on findings in our previous study, where we found an association between long-term prenatal paracetamol exposure and differences in DNAm in children with ADHD, using the Infinium 450 k platform [21]. Surprisingly, analyses of the follow-up data consisting of a larger sample and use of the Infinium EPIC platform have not replicated the results from our original study. Indeed, a challenge of EWASs is to discern spurious findings from true positives, rendering the replication of significant associations challenging [1, 22, 23]. Recent studies have shown low concordance across 450 k and EPIC platforms in different tissues [31, 32, 36–40]. Therefore, we have conducted a systematic evaluation of technical aspects related to concordance of DNAm data across the Infinium platforms in our studies in cord blood, by using data from a subset of samples with repeated measurements from the 450 k and EPIC platforms.

Technical variation such as batch effects is systematic variation caused by, for example, processing by different technicians, varying reagent batches and differences in the scanner performance. PCA of DNAm data from the samples with repeated measurements demonstrated distinct clustering of samples corresponding to the platform. If these differences in DNAm were independent of the platform and resulted entirely from positioning on the beadchip or bisulphite conversion plate, we would expect the changes to be relative and to not impact the replicability. Considering the general challenge of replication of EWASs [1, 22, 23] and the low per-CpG concordance across platforms reported in several recent studies [31, 32, 36–40], we were encouraged to examine possible cross-platform differences in DNAm. Corroborating previous studies, we observed a high per-sample correlation even between the randomly paired samples [32, 36–40]. In contrast, the per-CpG correlation was significantly lower, and some probes exhibited large differences in mean measured DNAm for overlapping CpGs on the two platforms.

Considering the highly concerning findings by Sugden et al*.* [31], reporting low reliabilities (measured by ICCs) for most CpGs across the 450 k and EPIC platforms in adult whole-blood, we estimated the ICCs of each CpG across the two platforms in our cord blood samples. Ideally, the ICC will approach 1 if the between-sample variation is much larger than the within-sample variation, suggesting larger biological variation than technical variation. However, most CpGs in our study exhibited poor reliabilities (ICC < 0.5) [31, 48] and we found that only 26.7% of CpGs in cord blood had an acceptable reliability across platforms. Interestingly, 38.6% of these CpGs overlapped with the 18.0% reliable CpGs identified in adult whole-blood [31]. This may suggest that some probes are generally unreliable in different tissues, possibly due to cell-type specific variability in DNAm. In contrast, other CpGs may perform worse in specific tissues, similar to what has been suggested for both per-CpG correlations and differences in DNAm between platforms [37–39]. In future studies, it would be interesting to examine the ICCs between Infinium platforms and other DNAm measuring technologies, such as whole-genome bisulphite sequencing (WGBS) or methylated immunoprecipitation (MeDIP).

We observed a substantial difference in the distribution of ICCs for different pre-processing steps used in common analysis pipelines. The *ENmix* pipeline exhibited the largest median ICC, suggesting that this pipeline may be better to best conserve the similarity of normalised repeated measurements from different platforms. In contrast, both the default *RnBeads*, *minfi* and *wateRmelon* pipelines have no better ICC distributions than the raw data. Notably, compared to a recent study reporting the ICC distribution of multiple different pipelines for within-platform repeated measurements [35], the distribution of cross-platform ICCs varies more dependent on the analysis pipeline used. However, the analysis pipeline with the highest median ICC is *ENmix* for both cross-platform and within-platform comparisons [35].

Interestingly, some studies have reported that cross-platform differences in DNAm and poor per-CpG correlations do not substantially impact the outcome of EWASs [32, 37]. However, when investigating the relationship of ICCs with the likelihood of replication of CpGs, Sugden et al*.* observed a positive relationship between increasing ICC and increasing replication rate for the association of DNAm with smoking [31]. Similar associations of ICCs with replicability have been found when ICCs were estimated from 450 k-450 k replicates [26, 49]. For instance, smoking-DNAm associations in whole-blood are highly replicable [56], and in one study, 96% of CpGs associated with smoking exhibit high reliability [26]. Additionally, poor ICCs have been shown to decrease the power of individual CpGs in EWASs, i.e. reducing the positive predictive value (PPV) by decreasing the number of true positives [28, 31, 47]. The median ICC of the significant CpGs in our original study was poor. However, if these findings were explained by the low reliability of the probes, we would expect none or very few significant CpGs. Consequently, based on the calculated ICCs using our 17 samples with repeated measurements, we have no explanation for the lack of replicability of our original findings.

A limitation of the present study is the small sample size used to assess the ICCs. However, ICC calculations generally require relatively small sample sizes [47, 57], and Sugden et al*.* found that sample sizes as small as 25 would be sufficient to detect 80% of all CpGs with an ICC ≥ 0.75 [31]. Furthermore, our results on both per-CpG correlations, differences in mean DNAm and ICCs are in line with other studies reporting one or more of these measurements for various tissues [31, 32, 36–40]. Nevertheless, a study including a larger number of repeated measurements in cord blood across the 450 k and EPIC platforms should be performed to strengthen our findings. Another limitation of our study is our inability to assess which technical variable(s) associated with the platform are contributing to the differences between platforms. Firstly, the DNAm on the 450 k and EPIC platforms was measured three years apart. Yet, this largely emulates the setting in most studies relying on data processed at different times and in different facilities (e.g., longitudinal studies and meta-analyses). Furthermore, all samples included in the current study were processed in the same core facility and by the same technician. Secondly, batches of bisulphite conversion reagents and scanners may also contribute to the cross-platform differences. Nevertheless, we expect that such technical variation is relative within the platforms and, consequently, that probes are mainly affected equally within the platform. Finally, it is challenging to assess the potential contribution of sample plate and beadchip to cross-platform differences, due to the different platform layouts (the 450 k beadchip can load 12 samples, while the EPIC beadchip can load 8 samples). To limit the contribution of variation from sample plate and beadchip in our data, the samples were randomly positioned on plates and beadchips. Accordingly, technical variation contributed by these variables should be random and should not inflict much bias when comparing DNAm between platforms.

The substantial differences across platforms revealed in this and previous studies [31, 32, 36–40] are troubling when trying to replicate findings using a different platform than in the original study. Replication of findings has long been considered a major challenge within epigenetic epidemiology [1, 22, 23], and to our knowledge, only one study has highlighted the potential impact of unreliable CpGs for replication of findings using data from different microarray platforms [31]. Challenges associated with differences in mean DNAm levels across platforms are not necessarily limited to issues of replication. For instance, longitudinal studies based on DNAm measured at multiple timepoints may suffer under the development of new microarray technologies (e.g., [41, 42]). Furthermore, this is also relevant for large meta-analyses combining data from multiple cohorts to increase the power of EWASs (e.g. [43, 44]), often based on large consortia such as the Pregnancy And Childhood Epigenetics (PACE) consortium [45]. Such strategies may be impacted by unreliable probes when combining data sets from different platforms. Similarly, unreliable CpGs across platforms may have implications for current EWAS knowledgebases, such as the EWAS Atlas [58] and the EWAS catalogue [59], which curate EWAS publications to report DNAm-trait associations.

## Conclusion

In conclusion, our failure to replicate significant CpGs associated with prenatal paracetamol exposure prompted a thorough investigation of potential technical origins of our null findings. The observation of low cross-platform per-CpG correlation and reliability corroborates previous reports. However, the low-reliability probes could not explain the inability to replicate previous findings in our case. Nevertheless, the poor cross-platform reliabilities may have important implications for future EWASs, in replication, meta-analyses and longitudinal studies. Therefore, we encourage researchers performing EWASs to examine the reliability of probes within and across tissues and to establish which probes are most comparable across microarray platforms. However, in many cases, the availability of repeated measurements from individual samples may be limited for reasons such as extra cost and limited availability of sample material. To this end, we encourage joint efforts to more extensively outline reliable probes in different tissues. If such investigations reveal common poor-performing probes across or within tissues, other studies may rely on this information when performing cross-platform studies. We hope our findings, supporting the results by Sugden et al*.* [31], increase awareness of possible challenges in including both 450 k and EPIC data in the same study. Cognisance and transparency of these challenges as well as appropriate precautions when performing cross-platform epigenetic investigations, may enhance the interpretation, replicability and validity of results, and could ultimately improve the clinical relevance of epigenetic epidemiology.

## Methods

### Sample population

We analysed cord blood samples from the Mother, Father and Child Cohort Study (MoBa). MoBa is a population-based pregnancy cohort study conducted by the Norwegian Institute of Public Health (NIPH) [60–63]. Participants were recruited from all over Norway from 1999–2008 [60, 61]. The women consented to participation in 41% of the pregnancies [60, 61]. The cohort includes approximately 114,500 children, 95,200 mothers and 75,200 fathers [60, 61]. The current study is based on Data Version 8 of the quality-assured data files released for research in 2015. Observational data from MoBa questionnaires Q1 (gestational week 0–13), Q3 (gestational week 13–29) and Q4 (gestational week 30 to delivery) were used to select individuals for the study. The personal, 11-digit identification number, unique to every permanent resident of Norway, was used to link MoBa to the Norwegian Patient Registry (NPR) and the Medical Birth Registry of Norway (MBRN). MBRN is a national health registry containing information about all births in Norway. We also analysed umbilical cord blood samples retrieved from the MoBa biobank [62, 63]. The biobank stores blood samples obtained from both parents during pregnancy, and from mothers and children (umbilical cord) at birth [62, 63].

The establishment of MoBa and initial data collection was based on a license from the Norwegian Data Protection Agency and approval from the Regional Committees for Medical and Health Research Ethics (REC). MoBa is currently regulated by the Norwegian Health Registry Act. All MoBa participants have given their written informed consent to participate in the cohort study. The current study has been approved by REC South East Norway (REC reference: 23,136, 2014/163). All data are de-identified, and the linkage between MoBa and the different health registries was handled by NIPH along with the relevant registries.

### Study design and measurements

The MoBa biobank contains 90,000 cord blood samples drawn at birth [63]. In our original study using the 450 k platform, we selected 384 samples from the biobank, and in the study using the EPIC platform, we selected 261 samples. Out of these samples, 611 samples were unique to either the 450 k data set or the EPIC data set, and 17 samples were measured on both the 450 k and EPIC platforms. The samples were selected based on prenatal exposure to paracetamol and child ADHD diagnosis, and all samples were term births (≥ 37 weeks). The 17 samples available in both data sets were all prenatally long-term exposed to paracetamol and had received an ADHD diagnosis.

Long-term prenatal exposure to paracetamol (Anatomical Therapeutic Chemical [ATC] code: N02BE01) was defined as the use of paracetamol for ≥ 20 days during pregnancy (coded as “yes” or “no”), based on a threshold from previous studies [64–68]. Use was self-reported and collected from three MoBa questionnaires (Q1, Q3 and Q4). Offspring diagnosis of ADHD was retrieved from the NPR (2008), containing all individual diagnoses asserted by specialists according to the 10^th^ revision of the International Classification of Disease (ICD-10), as reported by governmental hospitals and outpatient clinics. Children were defined as having ADHD if they had received an ICD-10 diagnosis of hyperkinetic disorder (HKD; F90.0, F90.1, F90.8 or F90.9) between 2008 and 2016. HKD corresponds to ADHD in the Diagnostic and Statistical Manual (DSM) system [69–72], as an HKD diagnosis requires both inattentiveness and hyperactivity symptoms.

### DNA methylation

#### Generation of DNAm data

The 450 k DNAm data from the samples in our original study are described elsewhere [21]. The samples assessed on the Infinium HumanMethylation EPIC BeadChip (Illumina) were processed similar to the 450 k data set [21]. Samples were randomly allocated to sample plates and beadchips, as previously described [21].

#### Quality control and pre-processing

Analyses were performed in the R programming language (http://www.r-project.org/). Quality control, normalisation and filtering of the data (Table 1) were performed using the default pipeline of *ENmix* [34]. The EPIC and 450 k data sets were pre-processed separately and all samples were included in the pre-processing (*n*_EPIC_ = 261; *n*_450k_ = 384). Subsequently, the 17 samples with repeated, cross-platform measurements were used for further analyses.

**Table 1:** Overview of retained probes upon filtering of data from the EPIC and 450 k microarray platforms

	EPIC probes	450 k probes
Raw data	866,091	485,512
> 5% low-quality values	857,144	479,914
SNP-enriched probe removal	827,968	463,111
Cross-reactive probe removal	813,047	441,548
Sex chromosome removal	795,515	431,536

First, samples with > 5% low-quality CpGs or low bisulphite intensity were removed (7 samples from the 450 k data set and 0 samples from the EPIC data set). Then, CpGs with > 5% low-quality values were also removed (5598 and 8947 CpGs from the 450 k and EPIC data sets, respectively). Background correction was performed using the *ENmix* exponential-truncated-normal out-of-band (oob) method [34], dye bias correction was executed using RELIC (REgression on Logarithm of Internal Control probes) [73], and probe-type correction was achieved using RCP (Regression of Correlated Probes) [74]. We removed probes with SNPs overlapping with the CpG interrogation site or the nucleotide extension site (*n*_EPIC_ = 29,176; *n*_450k_ = 16,803), cross-reactive probes (*n*_EPIC_ = 14,921; *n*_450k_ = 21,563) [36, 75–77] and probes on the sex chromosomes (*n*_EPIC_ = 17,532; *n*_450k_ = 10,012). These pre-processing steps resulted in a total of 795,515 probes in the EPIC data set and 431,536 probes in the 450 k data set. Of these, 397,813 CpGs overlapped between the two platforms.

#### Pre-processing using the default settings of common analysis pipelines

The raw data were also pre-processed using the default settings of four other common EWAS analysis pipelines: *ChAMP* [50, 51], *minfi* [52], *RnBeads* [53] and *wateRmelon* [54]. Additionally, we used the default *RnBeads* pipeline [53], but changed the background and probe-type correction methods to *Enmix.oob* [34] and BMIQ [55], respectively. The CpGs were annotated based on ilm10b4.hg19 [78].

### Statistical analyses

The *β* values (the ratio of methylated signal to the sum of methylated and unmethylated signal) were used for visualisations and calculation of all concordance measurements. To test for differences in distributions, we used the Kolmogorov–Smirnov test, and to test for differences in proportions, we used the Pearson’s Chi-squared test. To examine the correlations between both samples and CpGs from the different microarrays, we estimated the Pearson’s correlation coefficient. The ICC of each CpG was computed using the *irr* package [79]. We estimated the ICC by fitting an absolute agreement and mean of *k* raters (*k* = 2), two-way random effects model, as has previously been suggested for such comparisons [31]. The visualisation of the overlaps between studies of CpGs with mean DNAm differences > 0.5 across platforms was generated using the *UpSetR* package [80]

## Supplementary Information

**Additional file 1:** **Fig. S1.** Heatmap and clustering of genotyping probes. **Fig. S2.** Overlap of the CpGs exhibiting differences in mean DNAm > 0.5 in five studies. **Fig. S3.** Probe types and density distribution of Pearson’s and intra-class correlation coefficients. **Fig. S4.** Annotation groups and relation to Pearson’s and intra-class correlation coefficient categories. **Fig. S5.** Histogram of the distribution of the per-CpG island intra-class correlation coefficients. **Fig. S6.** Density plot of DNAm levels stratified by annotation categories. **Fig S7.** Histograms of the distribution of intra-class correlation coefficients for randomly paired samples. **Fig S8.** Scatter plot of the difference in mean DNAm level against the intra-class correlation coefficient. **Fig S9.** ICC distributions for the significant CpGs of our original study. **Table S1.** Overview of common pipelines with default settings for analysing DNA methylation data.

**Additional file 2:** Table of CpGs with corresponding intra-class correlation coefficients, Pearson’s correlation coefficients, mean difference across platforms, and whether the CpG exhibited an ICC ≥ 0.5 both in the current study and in the Sugden et al. study.

## Data Availability

The data that support the findings of this study are available from the Norwegian Mother, Father and Child Cohort Study, but restrictions apply to the availability of these data and so are not publicly available. However, data are available from the authors upon reasonable request and with permission from the Norwegian Mother, Father and Child Cohort Study.

## Abbreviations

ADHD: Attention deficit/hyperactivity disorder
ATC: Anatomical therapeutic chemical
DSM: Diagnostic and statistical manual
CpG: 5′–Cytosine–phosphate–guanine–3′ site
DNAm: DNA methylation
EWAS: Epigenome-wide association study
FDR: False discovery rate
HKD: Hyperkinetic disorders
ICC: Intra-class correlation coefficient
ICD-10: The 10th revision of the International Classification of Disease
MBRN: The Medical Birth Registry of Norway
MoBa: The Norwegian Mother, Father and Child Cohort Study
NIPH: The Norwegian Institute for Public Health
NPR: The Norwegian Patient Registry
PCA: Principal component analysis
PPV: Positive predictive value
REC: The Regional Committees for Medical and Health Research Ethics
