# Supplementary material for: Low reliability of DNA methylation across Illumina Infinium platforms in cord blood: implications for replication studies and meta-analyses of prenatal exposures
Source: Clin Epigenetics. 2022 Jun 28;14:80. doi: 10.1186/s13148-022-01299-3 (PMC9238140; doi:10.1186/s13148-022-01299-3)
Supplement: Supplementary file 1 — Additional file 1: Fig. S1. Heatmap and clustering of genotyping probes. Fig. S2. Overlap of the CpGs exhibiting differences in mean DNAm > 0.5 in five studies. Fig. S3. Probe types and density distribution of Pearson’s and intra-class correlation coefficients. Fig. S4. Annotation groups and relation to Pearson’s and intra-class correlation coefficient categories. Fig. S5. Histogram of the distribution of the per-CpG island intra-class correlation coefficients. Fig. S6. Density plot of DNAm levels stratified by annotation categories. Fig S7. Histograms of the distribution of intra-class correlation coefficients for randomly paired samples. Fig S8. Scatter plot of the difference in mean DNAm level against the intra-class correlation coefficient. Fig S9. ICC distributions for the significant CpGs of our original study. Table S1. Overview of common pipelines with default settings for analysing DNA methylation data. [file 13148_2022_1299_MOESM1_ESM.docx]

**Supplementary Material**

**Additional file 1 (.docx)**

**Figure S1.** Heatmap and clustering of genotyping probes.

**Figure S2.** Overlap of the CpGs exhibiting differences in mean DNAm >0.5 in five studies.

**Figure S3.** Probe types and density distribution of Pearson’s and intra-class correlation coefficients.

**Figure S4.** Annotation groups and relation to Pearson’s and intra-class correlation coefficient categories.

**Figure S5.** Histogram of the distribution of the per-CpG island intra-class correlation coefficients.

**Figure S6.** Density plot of DNAm levels stratified by annotation categories.

**Figure S7.** Histograms of the distribution of intra-class correlation coefficients for randomly paired samples.

**Figure S8.** Scatter plot of the difference in mean DNAm level against the intra-class correlation coefficient.

**Figure S9.** ICC distributions for the significant CpGs of our original study.

**Table S1.** Overview of common pipelines with default settings for analysing DNA methylation data.


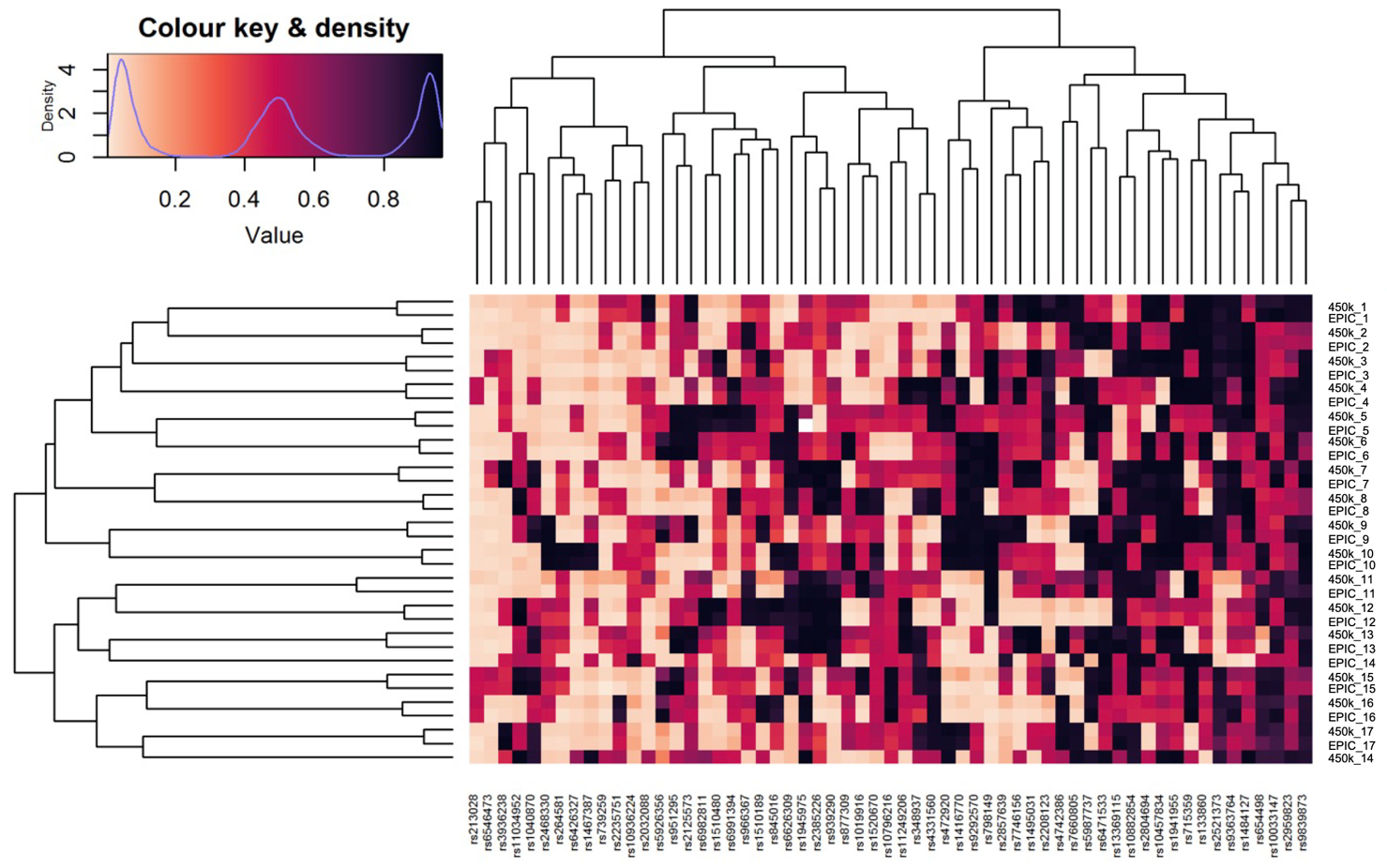


***Figure S1.*** *Heatmap and clustering of the 17 samples from the 450k and EPIC platforms, based on the 59 common genotype probes. The samples mostly cluster together in corresponding pairs, suggesting that no samples have been mixed up.*

*
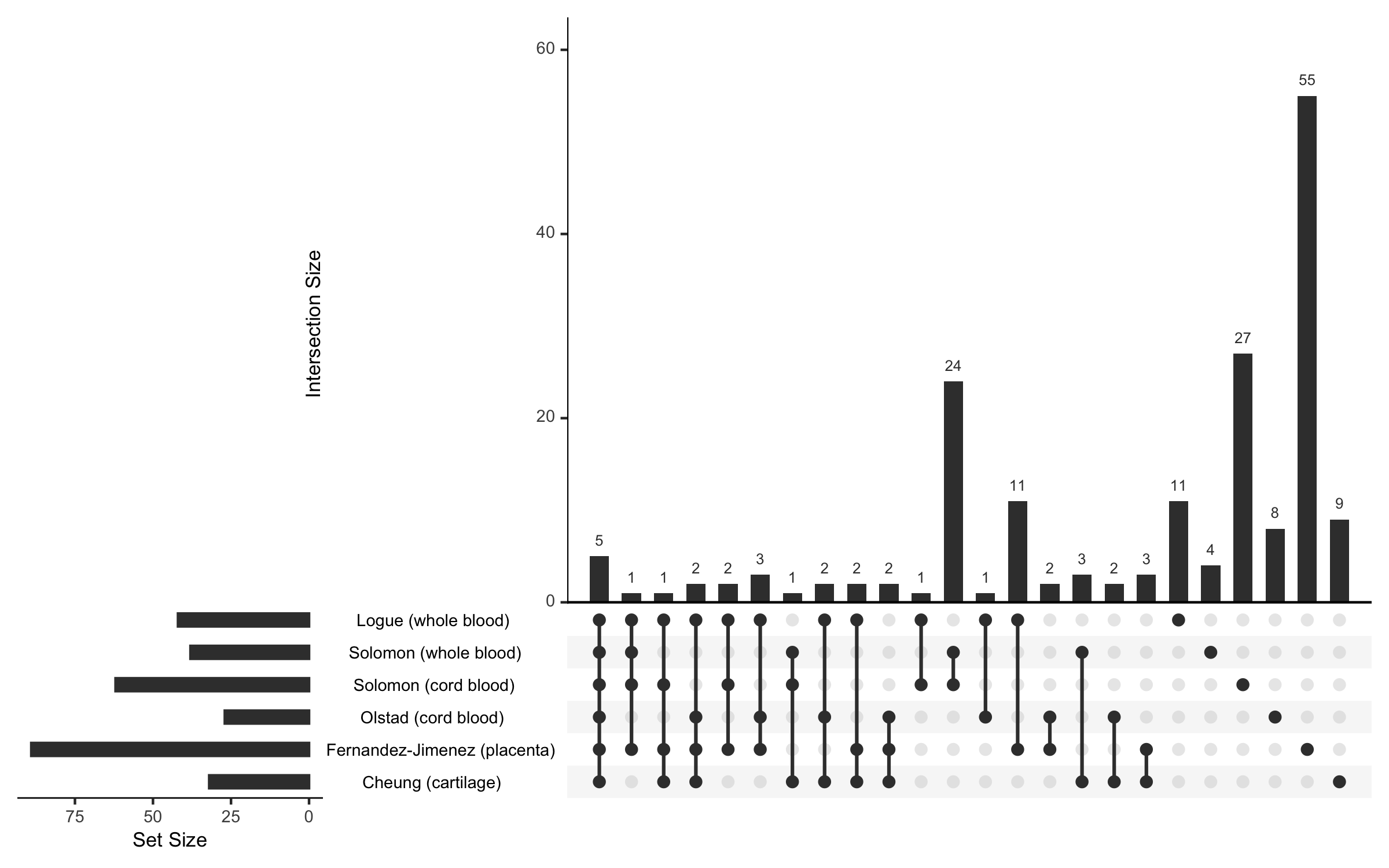
*

***Figure S2.*** *Overlap of the CpGs exhibiting differences in mean DNAm (*Δβ*) >0.5 in the five studies reporting these measures across the 450k and EPIC platforms including the current study, performed in whole or cord blood* [1, 2]*, the placenta* [3] *or cartilage* [4]*. The “Set Size” is the number of CpGs with mean* Δβ *>0.5 in each study, the filled dots indicate whether CpGs from a specific study participates in the overlapping set of CpGs, and the bar plot indicates the number of CpGs overlapping, for the particular intersected set. E.g., of the CpGs with a mean* Δβ *>0.5, 5 are common across all studies (first bar, indicated with a filled dot for all studies).*

##
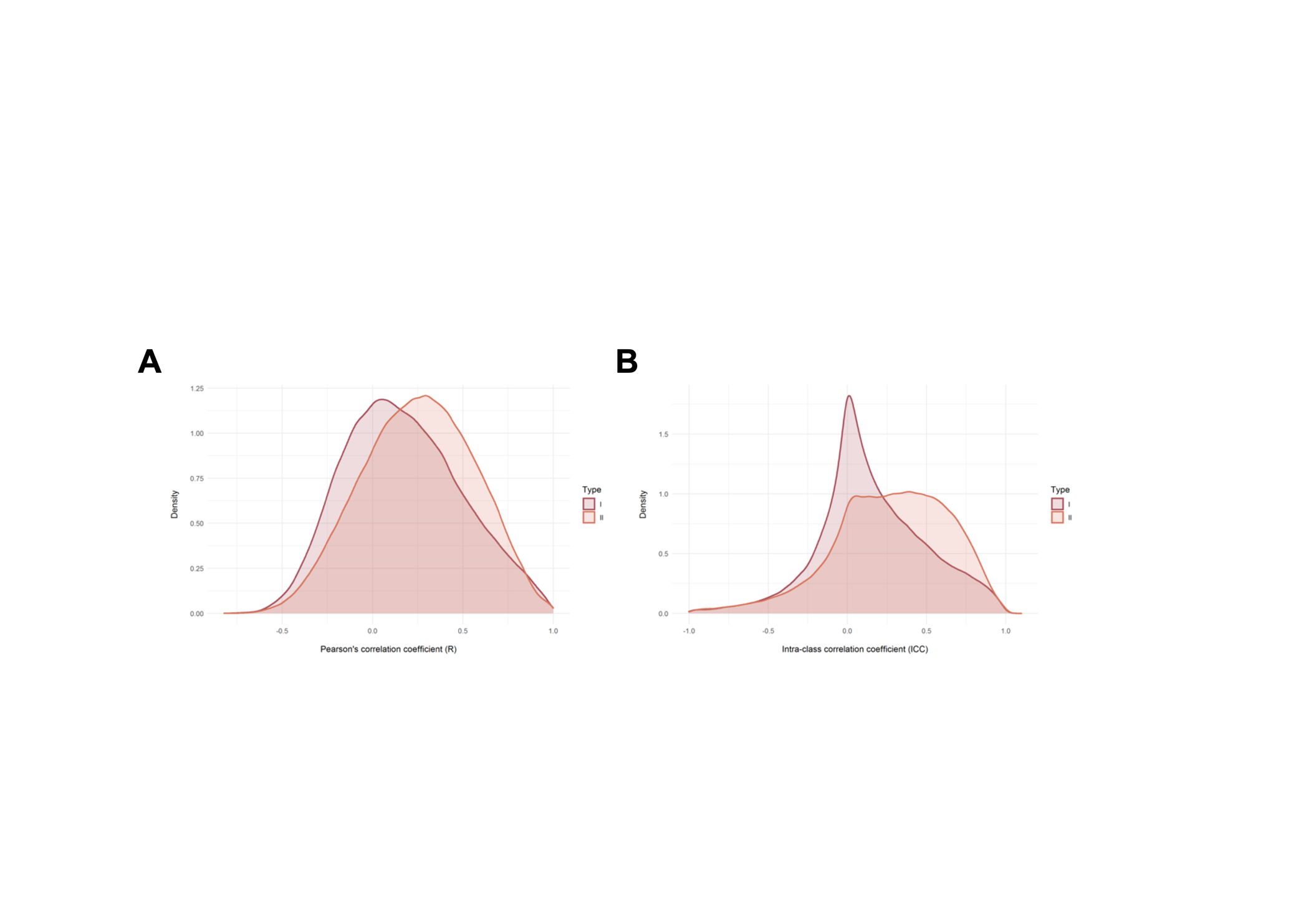


***Figure S3.*** *Density plot of per-probe* ***(A)*** *Pearson’s correlation coefficients and* ***(B)*** *intra-class correlation coefficients (ICC), stratified by microarray probe types. Outlying CpGs with ICCs less than the mean ICC minus three standard deviations, were removed for visualisation purposes, but were included for summary statistic calculations.*

*
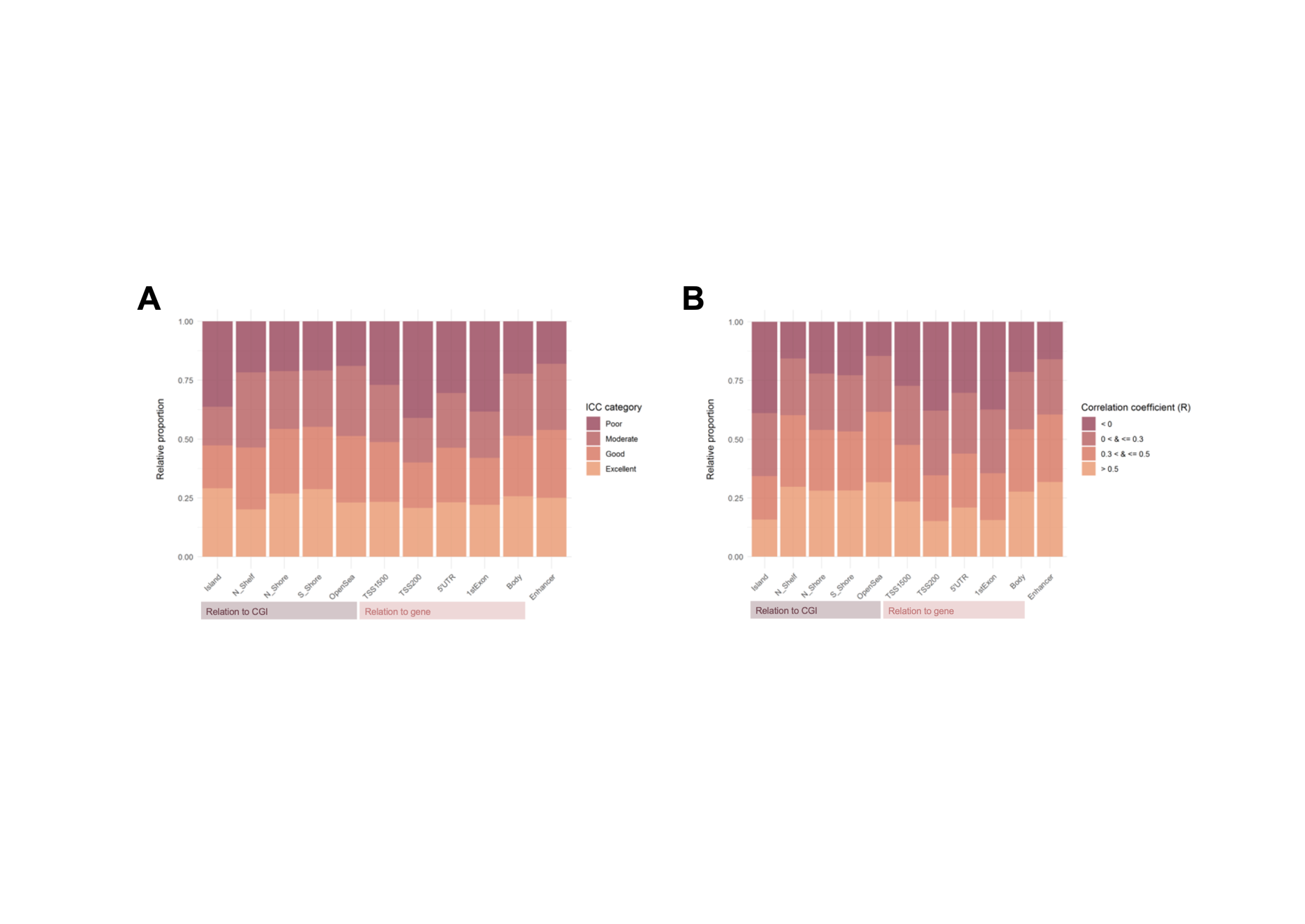
*

***Figure S4.*** *Stacked bar plots of the distribution of the CpGs’ relations to CpG islands (CGIs) and genes. The frequencies account for the number of CpGs within each* ***(A)*** *ICC category or* ***(B)*** *correlation coefficient category, to make the proportions comparable.* ***Abbreviations:*** *N_Shelf: “north” shelf; N_Shore: “north” shore; S_Shore: “south” shore; TSS: transcription start site; 5’UTR: 5’ untranslated region.*


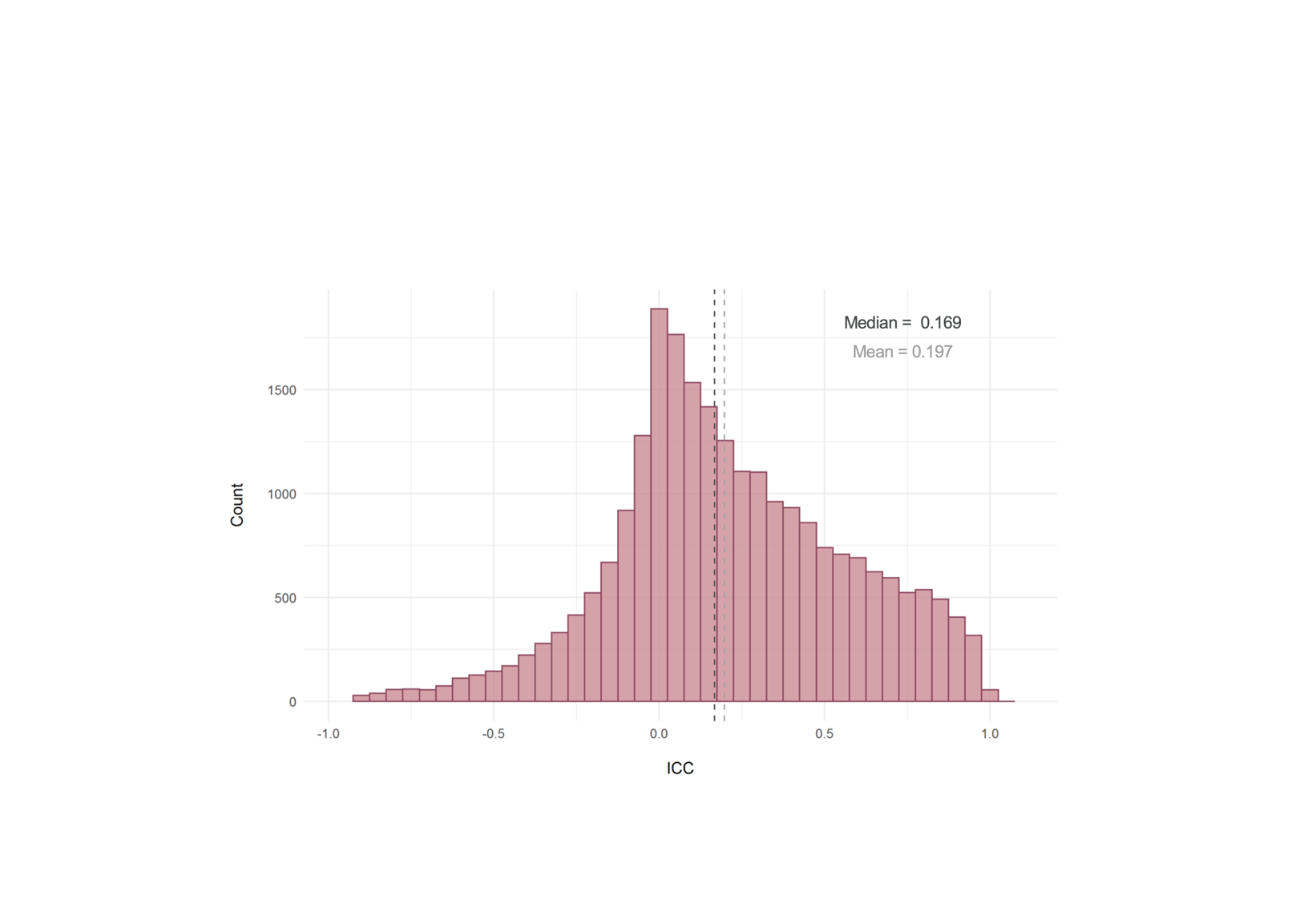


***Figure S5.*** *Histogram of the distribution of intra-class correlation coefficients (ICCs), based on the mean DNAm values of each CpG island (*n *= 24,231 islands). The dark grey, dotted line indicates the median ICC, and the light grey, dotted line indicates the mean ICC. Outlying CpGs with ICCs less than the mean ICC minus three standard deviations, were removed for visualisation purposes, but were included for summary statistic calculations.*

*
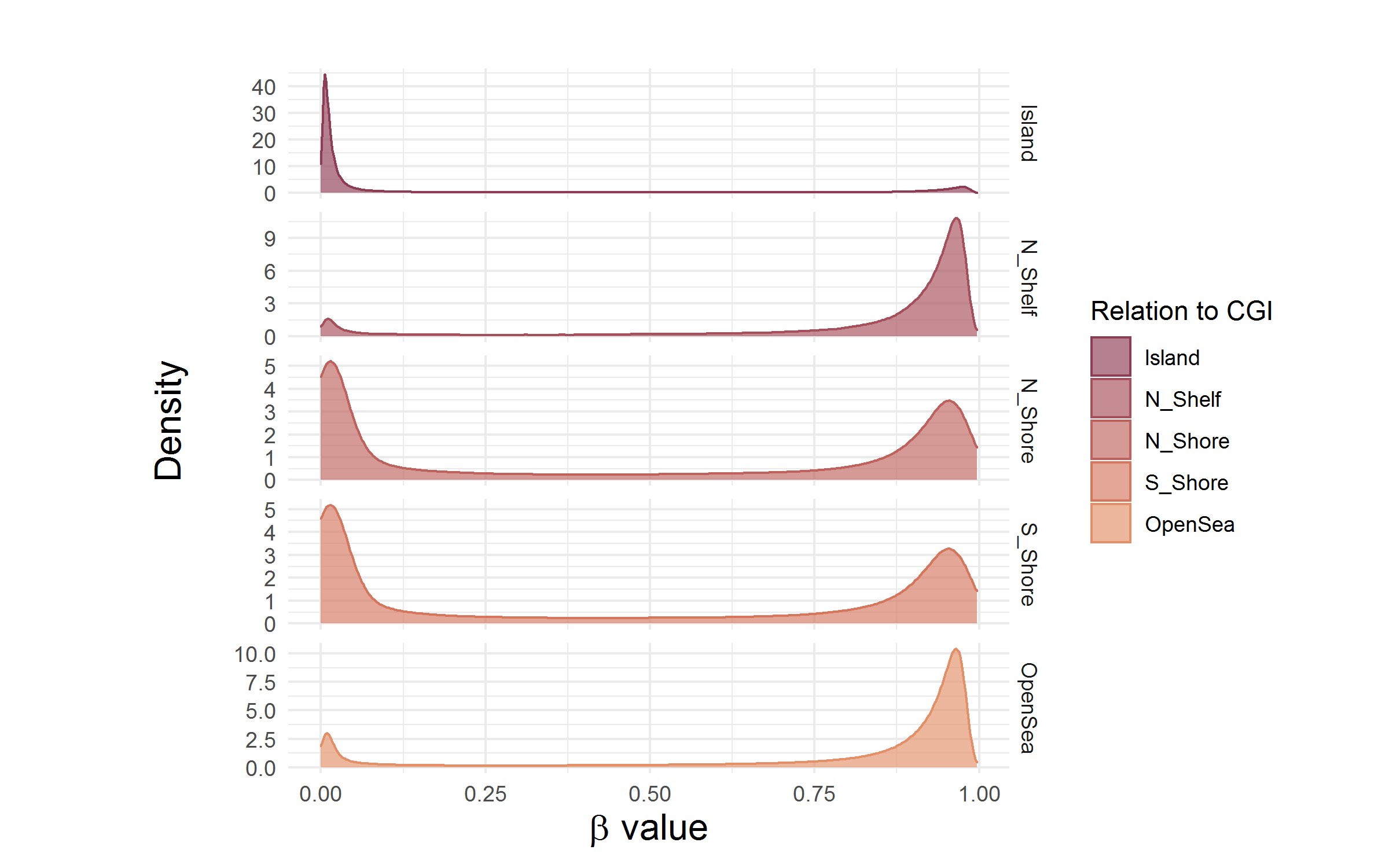
*

***Figure S6.*** *Density plots of DNAm levels (*β *values), stratified by the CpGs’ relation to CpG islands (CGIs).* ***Abbreviations:*** *N_Shelf: “north” shelf; N_Shore: “north” shore; S_Shore: “south” shore; TSS: transcription start site; 5’UTR: 5’ untranslated region.*

*
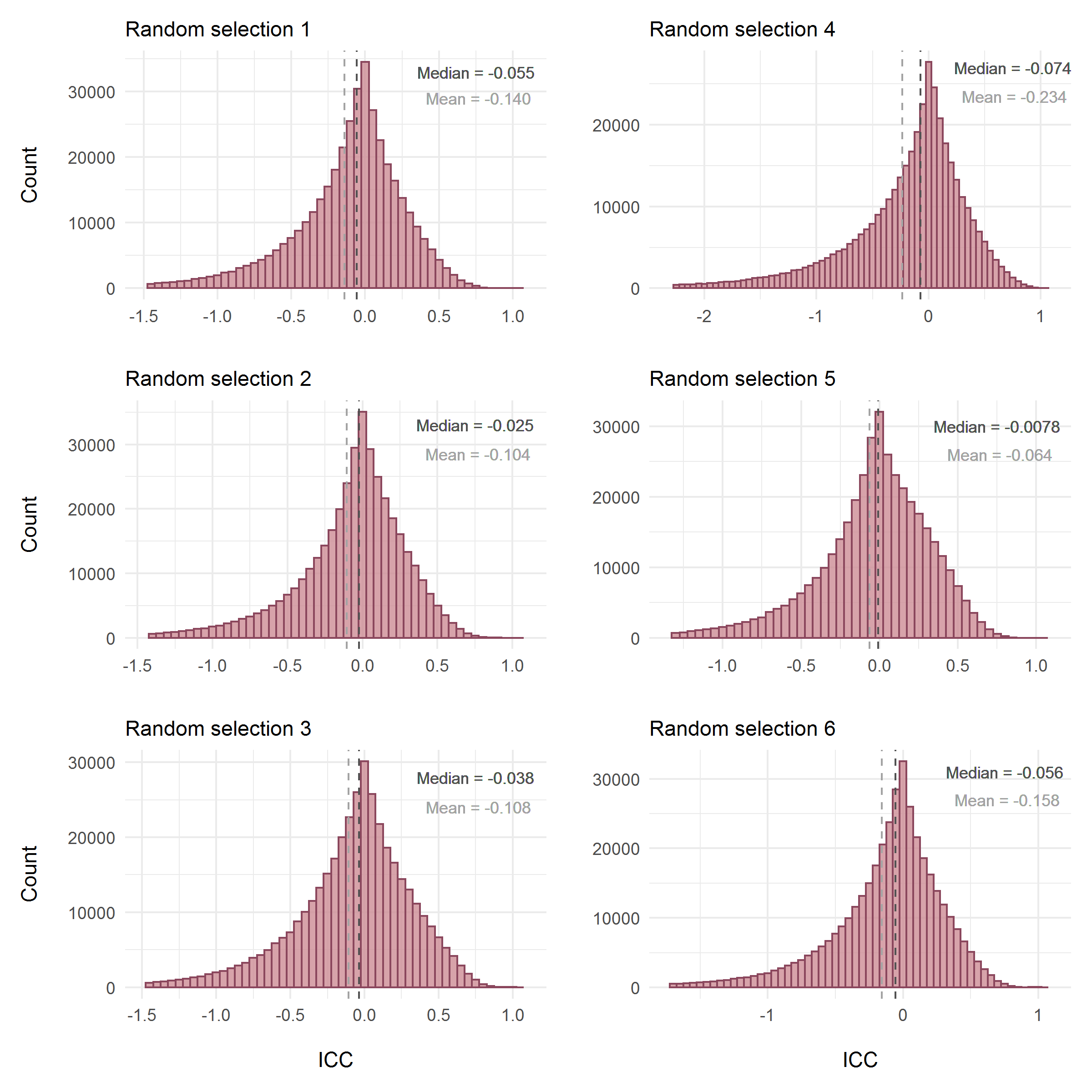
*

***Figure S7.*** *Histograms of the distribution of intra-class correlation coefficients (ICCs), comparing the 17 EPIC samples randomly paired with a 450k sample. Six random EPIC-450k combinations were drawn. The dark grey, dotted line indicates the median ICC, and the light grey, dotted line indicates the mean ICC. Outlying CpGs with ICCs less than the mean ICC minus three standard deviations, were removed for visualisation purposes, but were included for summary statistic calculations.*

*
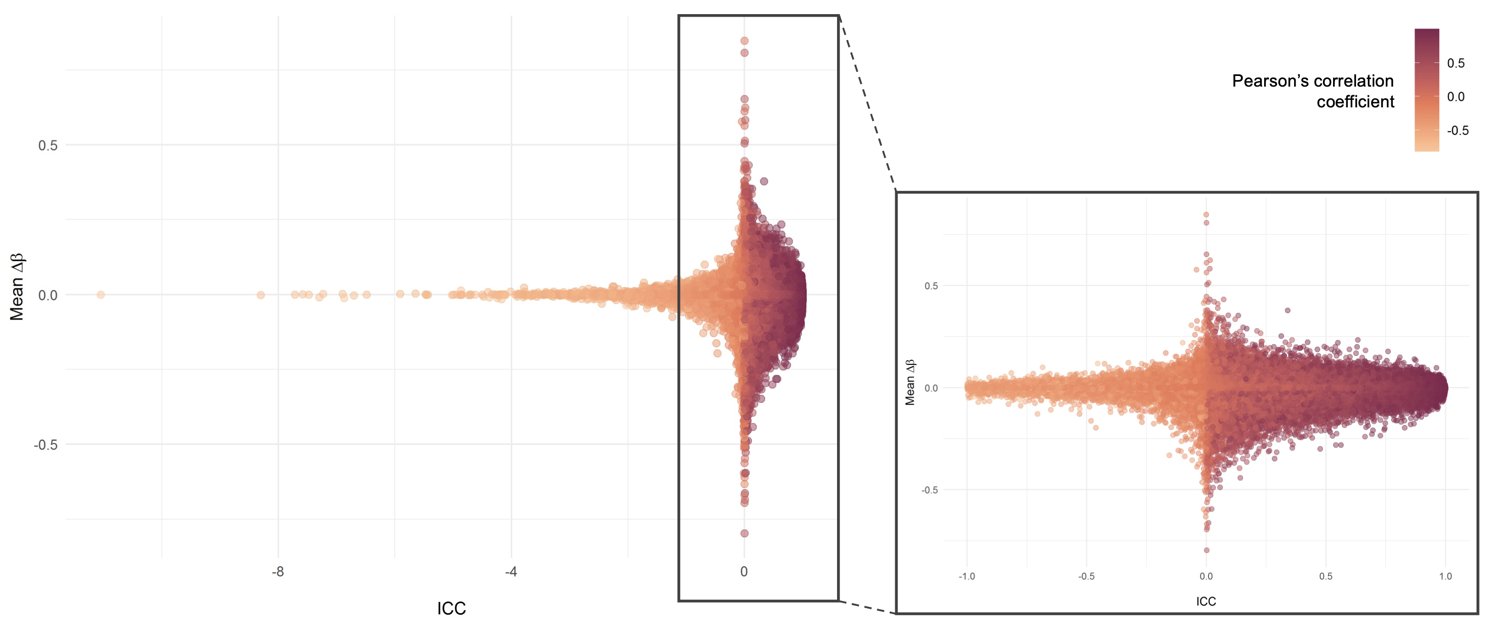
*

***Figure S8.*** *Scatter plot of the difference in mean DNAm level (*Δβ*) against the intra-class correlation coefficient (ICC), calculated from the repeated measurements on the 450k and EPIC platforms. Each point corresponds to one CpG, and are coloured according to the Pearson’s correlation coefficient of the CpG between the 450k and EPIC platforms.*

*
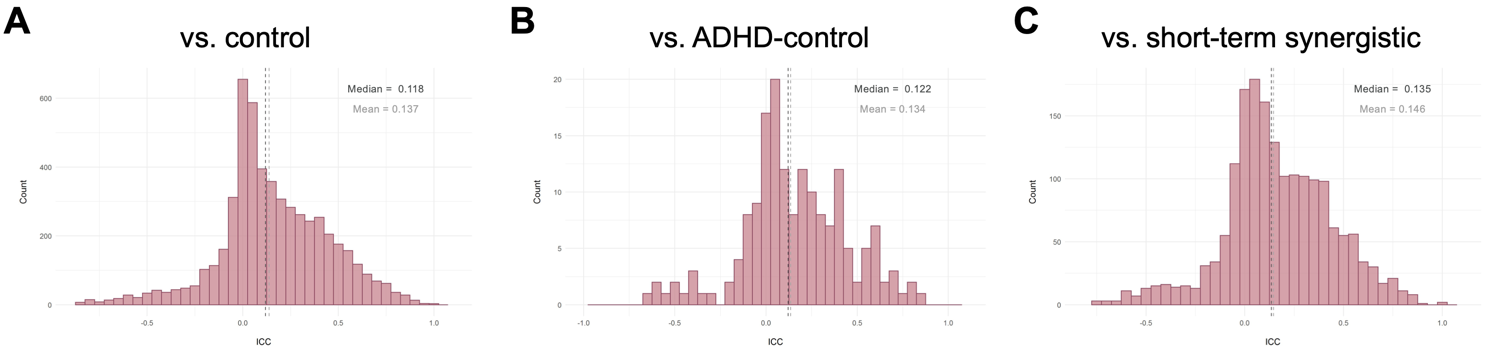
*

***Figure S9.*** *ICC distributions for the significant CpGs of our original study* [5]*, when comparing the prenatally long-term paracetamol exposed group with ADHD to* ***(A)*** *the control group (*n *= 5436 CpGs),* ***(B)*** *ADHD control group (*n *= 171 CpGs), or* ***(C)*** *short-term paracetamol exposed group with ADHD (“short-term synergistic”;* n *= 1,811 CpGs). The dark grey, dotted line indicates the median ICC, and the light grey, dotted line indicates the mean ICC. Outlying CpGs with ICCs less than the mean ICC minus three standard deviations, were removed for visualisation purposes, but were included for summary statistic calculation.*

**Table S1.** Overview of common pipelines with default settings for analysing DNA methylation data.

|  | ***ChAMP*** | ***ENmix**** | ***minfi***** | ***RnBeads*** | ***RnBeads (customised)*** | ***wateRmelon*** |
| --- | --- | --- | --- | --- | --- | --- |
|  | *Filtering* | | | | | |
| **Time of filtering** | Before normalisation | Before normalisation | After normalisation  (except for removal of poor *samples* before normalisation) | Before, and after normalisation^e^ | Before, and after normalisation^b^ | Before normalisation |
| **Samples^a^ removed** | - Samples with >10% missing DNAm values - Outliers^b^ | - Samples with >10% missing DNAm values - Poor samples - Outliers^c^ | - Poor samples (detection *p* value) | - Poor samples (Greedycut^f^) | - Poor samples (Greedycut^f^) | - Outliers^h^ - Poor samples (detection *p* value) |
| **Probes filtered** | - Poor probes (detection *p* value or bead count) - Non-CpG probes - All probes containing SNPs [6] - All multi-hit probes [7] - Probes on sex chromosomes | - Poor probes (detection *p* value or bead count) | - Poor probes (detection *p* value) - Probes with an SNP either on the CpG interrogation site or the single nucleotide extension site - Cross-reactive probes [8–11] - Probes on sex chromosomes | - SNP-enriched probes (last 3 bases of the probe overlaps with an SNP) - Poor probes (Greedycut^f^) - Context-specific probes (exclude non-CpG probes) - Probes on sex chromosomes | - SNP-enriched probes (last 3 bases of the probe overlaps with an SNP) - Cross-reactive probes - Poor probes (Greedycut^f^) - Context-specific probes (exclude non-CpG probes) - Probes on sex chromosomes | - Poor probes (detection *p* value or bead count) |
| **Def. of poor probes/samples** | Samples having >10% probes with a detection *p* value >0.01 and probes with a detection *p* value >0.01. Probes if bead count <3 for >5% of samples. | Samples and probes with >5% of values with a detection *p* value >0.000001 or bead count >3. Bisulphite intensities of <mean–3*s.d. of the bisulphite control intensities are also considered low-quality. | Detection *p* value >0.01 for either samples or probes. | Uses the Greedycut algorithm^f^. Samples or probes having >1% of sites with a detection p value >0.05. | Uses the Greedycut algorithm^f^. Samples or probes having >1% of sites with a detection p value >0.01. | Samples or probes having >1% of sites with a detection p value >0.05. Probes if bead count <3 for >5% of samples. |
|  | *Normalisation* | | | | | |
| **Background correction** | None | *ENmix oob* | None^d^ | *dasen*^g^ | *ENmix oob* | *dasen*^g^ |
| **Dye correction** | None | *RELIC* | None | None | None | None |
| **Probe-type bias correction** | BMIQ | RCP | Stratified quantile normalisation | *dasen*^g^ | BMIQ | *dasen*^g^ |

* The default settings cause “removal” of outliers and low-quality values by assigning them missing as defined by the ‘qcfilter()’ function. Consequently, the default settings will introduce many missing values in the data set (argument ‘qc’ is set to FALSE, while ‘fqcfilter’ is set to TRUE). However, if one set ‘qc’ to TRUE, this argument calls the ‘QCinfo()’ function, which *removes* low quality and low intensity samples and values before any outliers are removed. When this is done first, no outliers are identified for any of the data sets, and the number of missing values is significantly reduced. Therefore, to limit the number of missing values which may impact the calculated ICCs, we changed two of the default settings by setting ‘qc’ = TRUE and ‘fqcfilter’ = FALSE.

** Using the popular analysis pipeline described by Maximovich *et al.* [12], as it mainly uses the *minfi* package for pre-processing purposes. Currently, “The minfi User’s Guide” [13] is incomplete for the pre-processing steps.

^a^ None of the replicate samples were removed as an outlier or poor-performing sample in neither of the analysis pipelines.

^b^ Per-CpG values <3*IQR from the lower quartile, or >3*IQR from the upper quartile.

^c^ Defined as deviating total intensity (bisulphite intensities of <mean–3*s.d. of the bisulphite control intensities are considered low-quality values) or deviation from the *β* value distribution.

^d^ No background correction, but low outlying intensities are fixed.

^e^ Context-specific probes and probes on sex chromosomes are removed after normalisation.

^f^ Greedycut is an iterative algorithm consecutively removing the most impure samples and probes (i.e., the columns and rows containing the largest proportion of unreliable measurements [highest detection *p* value]).

^g^ The *dasen* method includes both background correction. Background is corrected by adding the intensity difference between probe types I and II, to the intensities of Type I probes (i.e., this method aims to equalize the background of the probes to minimize probe type differences). Additionally, *dasen* performs inter-array quantile normalisation, which results in the actual removal of background gradient from the raw intensities. This normalization is performed on the type I and II probes separately.

^h^ As defined by the default settings to the ‘outlyx()’ function implemented in the *wateRmelon* package.

**Abbreviations:** oob: out-of-band; s.d.: standard deviation; SNP: single nucleotide polymorphism.

**References**

1. Logue MW, Smith AK, Wolf EJ, Maniates H, Stone A, Schichman SA, et al. The correlation of methylation levels measured using Illumina 450K and EPIC BeadChips in blood samples. Epigenomics. Future Science Group; 2017;9:1363–71.

2. Solomon O, MacIsaac J, Quach H, Tindula G, Kobor MS, Huen K, et al. Comparison of DNA methylation measured by Illumina 450K and EPIC BeadChips in blood of newborns and 14-year-old children. Epigenetics. 2018;13:655–64.

3. Fernandez-Jimenez N, Allard C, Bouchard L, Perron P, Bustamante M, Bilbao JR, et al. Comparison of Illumina 450K and EPIC arrays in placental DNA methylation. Epigenetics. 2019;14:1177–82.

4. Cheung K, Burgers MJ, Young DA, Cockell S, Reynard LN. Correlation of Infinium HumanMethylation450K and MethylationEPIC BeadChip arrays in cartilage. Epigenetics. Taylor & Francis; 2020;15:594–603.

5. Gervin K, Nordeng H, Ystrom E, Reichborn-kjennerud T, Lyle R. Long-term prenatal exposure to paracetamol is associated with DNA methylation differences in children diagnosed with ADHD. Clin Epigenetics. 2017;9.

6. Zhou W, Laird PW, Shen H. Comprehensive characterization, annotation and innovative use of Infinium DNA methylation BeadChip probes. Nucleic Acids Res. Nucleic Acids Res; 2017;45:e22.

7. Nordlund J, Bäcklin CL, Wahlberg P, Busche S, Berglund EC, Eloranta ML, et al. Genome-wide signatures of differential DNA methylation in pediatric acute lymphoblastic leukemia. Genome Biol. BioMed Central; 2013;14:1–15.

8. Chen Y, Lemire M, Choufani S, Butcher DT, Grafodatskaya D, Zanke BW, et al. Discovery of cross-reactive probes and polymorphic CpGs in the Illumina Infinium HumanMethylation450 microarray. http://dx.doi.org/104161/epi23470. Taylor & Francis; 2013;8:203–9.

9. Benton MC, Johnstone A, Eccles D, Harmon B, Hayes MT, Lea RA, et al. An analysis of DNA methylation in human adipose tissue reveals differential modification of obesity genes before and after gastric bypass and weight loss. Genome Biol. BioMed Central; 2015;16:1–21.

10. McCartney DL, Walker RM, Morris SW, McIntosh AM, Porteous DJ, Evans KL. Identification of polymorphic and off-target probe binding sites on the Illumina Infinium MethylationEPIC BeadChip. Genomics Data. Elsevier Inc; 2016;9:22–4.

11. Pidsley R, Zotenko E, Peters TJ, Lawrence MG, Risbridger GP, Molloy P, et al. Critical evaluation of the Illumina MethylationEPIC BeadChip microarray for whole-genome DNA methylation profiling. Genome Biol. 2016;17.

12. Maksimovic J, Phipson B, Oshlack A. A cross-package Bioconductor workflow for analysing methylation array data. F1000Research. Faculty of 1000 Ltd; 2016;5.

13. Hansen KD, Fortin J-P. The minfi User’s Guide [Internet]. 2021.
